# Fixed Drug Eruption in an Epileptic Patient Previously Receiving Treatment With Phenytoin for Seven Years

**DOI:** 10.1177/2324709613512622

**Published:** 2013-11-19

**Authors:** Keaton S. Smetana, Katie J. Suda, Leslie A. Hamilton

**Affiliations:** 1 University of Tennessee Health Science Center College of Pharmacy, Memphis, TN, USA

**Keywords:** phenytoin, adverse drug reaction, administration rate, fixed drug eruption

## Abstract

A 52-year-old African American female presented with severe left thigh pain of unknown etiology. She had a past medical history of generalized seizure disorder treated with phenytoin for 7 years without incident. During admission a nurse witnessed a seizure, and consequently loading and maintenance doses of phenytoin were administered to obtain a therapeutic serum concentration. The patient had a history of noncompliance with multiple subtherapeutic phenytoin levels. Subsequently, unifocal blue discolored spots appeared, progressing to a bullous component that was positive for skin sloughing. Drug-induced fixed drug eruption was diagnosed and attributed to phenytoin. Clinicians should be cognizant of drug-induced fixed drug eruption in patients just initiated and those receiving long-term treatment with phenytoin. The administration rate of phenytoin may be associated with the development of fixed drug eruption.

## Introduction

Phenytoin is a hydantoin anticonvulsant indicated for status epilepticus, partial and generalized seizures, and adjunctive seizures treatment.^1^ Idiosyncratic drug-induced rashes are rare but have been associated with antiepileptic drugs (AED). The rash can be a maculopapular or erythematous pruritic rash that resolves on medication discontinuation. However, the rash can be life-threatening (Steven–Johnsons syndrome [SJS] or toxic epidermal necrolysis [TEN]).^2^ The only significant predictor associated with rash formation in AEDs is previous rash formation.^3^ When comparing rates of rash for AEDs, phenytoin, carbamazepine, and lamotrigine were found to have significantly higher incidences of dermatologic eruption, when compared to other AEDs.^2,3^

Phenytoin requires close monitoring secondary to narrow therapeutic index and nonlinear pharmacokinetics. Small dosage changes may result in a nonproportional increase in the steady-state concentration, which may lead to adverse events.^4^ The phenytoin boxed warning recommendation is not exceeding an intravenous administration rate of 50 mg/min secondary to severe hypotension and cardiac arrhythmias.^1^ Also, the rate at which the therapeutic concentration is reached may contribute to the development of drug eruptions.^5^

We describe an adult female patient with a history of generalized seizure disorder with subtherapeutic phenytoin levels, who experienced a fixed drug eruption (FDE) after rapidly reinitiating phenytoin. The patient was diagnosed with generalized seizure disorder 13 years prior and had previous admissions with undetectable phenytoin levels.

## Case Description

A 52-year-old black female was admitted for severe left thigh pain of unknown etiology. She was seen in the emergency department of the same institution 2 months prior secondary to left thigh pain “from a spider bite” and was diagnosed with a left buttock abscess. She was discharged with doxycycline 100 mg twice daily and sulfamethoxazole–trimethoprim 800 mg to 160 mg twice daily. On discharge, the patient reported that the pain became progressively worse with a self-reported pain rating of 10 on a 10-point scale. Relevant medical history included hypertension, type 2 diabetes, hyperlipidemia, asthma, gastroesophageal reflux, and generalized seizure disorder with the last reported seizure 4 months prior to the current admission. The patient denied any use of alcohol, tobacco, or illicit drugs. The patient denied drug allergies, but the medical record indicated allergies to levofloxacin, morphine, and penicillin. Her home medications were hydroxyzine 25 mg 3 times daily, albuterol inhaler as needed, insulin glargine 80 units at bedtime, insulin aspart 35 units with meals, amlodipine 10 mg daily, benazepril 20 mg daily, and phenytoin 100 mg twice daily.

On admission, a physical examination revealed pulses of 2 out of 4 in all extremities, but the patient was guarded on left thigh examination. Neurologic exam revealed bilateral numbness in both feet due to microvascular diabetic complications. Pelvis and thigh magnetic resonance imaging revealed myositis involving the vastus lateralis of her left leg with no evidence of osteomyelitis. Culture results were negative. Edema was also seen anterior, medial, and lateral to the left rectus femoris. Views of each tibia and fibula revealed no acute fractures or dislocations, normal alignment of the ankle mortise joint, and overlying soft tissues appeared unremarkable. Initial laboratory values in the emergency department revealed a glucose level of 561 mg/dL (normal = 70-130 mg/dL), urea nitrogen 10 mg/dL (8-20 mg/dL), creatinine 0.9 mg/dL (0.5-1.5 mg/dL), aspartate aminotransferase (AST) 23 U/L (5-30 U/L), albumin 3.2 g/dL (3.5-5 g/dL), white blood cells with differential within normal limits, erythrocyte sedimentation rate 63 (0-20 mm/h), and temperature 36.4°C. Inpatient medications included phenytoin 100 mg twice daily, amlodipine 10 mg daily, aspirin 81 mg daily, benazepril 20 mg daily, clindamycin 600 mg 3 times daily, heparin 5000 units 3 times daily, insulin aspart 15 units before meals, detemir 20 units twice daily, and human insulin 3 to 15 units sliding scale protocol before meals. Medications used as needed included albuterol inhaler, acetaminophen, and oxycodone. Clindamycin was discontinued on hospital day 2.

The day after admission, laboratory values were the following: glucose 142 mg/dL, urea nitrogen 3.6 mg/dL, creatinine 0.5 mg/dL, albumin 2.8 g/dL, AST 10 U/L, and phenytoin 1.9 µg/mL. The corrected phenytoin level was 3 µg/mL (normal = 10-20 µg/mL; albumin = 2.8 g/dL).^6^ At that time, no changes were made. Overnight, a nurse observed the patient having suspected seizure activity and noted left to right head movements, chewing of the tongue, right lower and upper extremity movements, and incontinence of urine. A 400-mg loading dose of oral phenytoin was administered for 4 doses over a total of 7 hours, for a total of 1600 mg (17 mg/kg), and a maintenance dose of phenytoin was initiated at 300 mg (3.2 mg/kg) at bedtime. The subsequent phenytoin level the following day was 13.4 mg/L with a corrected phenytoin level of 20 mg/L. After 2 days on 300 mg of phenytoin, a unifocal blue discolored spot on her thigh progressed to be multifocal areas that were mostly concentrated on her right thigh and lower abdomen. A skin biopsy of the affected area confirmed a FDE and phenytoin was discontinued and changed to levetiracetam 1000 mg twice daily. The following day a bullous component developed to the fixed drug reaction that progressed to rupturing and was positive for skin sloughing. Dermatology excluded SJS and TEN as possible diagnoses. Five days after the discontinuation of phenytoin, the rash was noted to be stable, healing well, and improving with zinc oxide ointment.

## Discussion

Generalized bullous FDE are characterized by multiple, large, purplish patches, at times with flaccid blisters.^7^ Lesions often appear symmetrical and often appear on the extremities, genitalia, and intertriginous sites. Mucosal sites are usually not involved and recovery is often rapid and complete without sequelae once the offending agent has been removed. Retrial of the offending drug will result in lesions occurring in the same spot as prior episodes, but may have greater involvement.^8^ As was evident in the case presented above, FDE usually has favorable outcomes once the offending agent is removed. It is important to differentiate FDE from SJS and TEN due to the life-threatening events associated with the later diagnoses.

Serum concentrations of phenytoin are possibly associated with the risk of developing drug-induced skin eruptions. In a prospective study, mean serum concentrations of phenytoin was high in patients who developed a rash.^5^ Chronic toxicity of phenytoin is not directly linked to skin eruptions, but early skin eruptions may be linked to an active intermediate metabolite. The major metabolite of phenytoin is probably not responsible for drug eruptions observed early in therapy because it would not be present in a high concentration due to lack of metabolism. If the intermediate metabolite was linked to drug-related skin eruptions, initiating therapy at a lower dose may produce lower incidences of rashes.^5^ An in vitro study found that intermediate metabolite accumulation was linked to the development of phenytoin-induced drug eruptions.^9^

In the patient case presented here, the outcome of the possible drug reaction may be secondary to the rate at which oral phenytoin was administered to obtain a therapeutic concentration and the presence of a metabolite. Use of the Naranjo adverse drug reaction probability scale indicated a probable relationship with a score of 5 for phenytoin and the development of an FDE.^10^ This case answered yes to the following questions on the Naranjo scale: previous reports, adverse event appeared after the phenytoin was administered, the adverse reaction improved on discontinuing phenytoin, and confirmed by objective evidence.^10^ Furthermore, the resolution of the symptoms after discontinuation of phenytoin supports the association in the development of the FDE. While the reaction may have been accelerated by uncontrolled diabetes, we believe that the myositis was less likely to have caused the FDE. Also, the myositis was present on the left thigh, with the FDE presenting on the right thigh. Also, clindamycin and sulfamethoxazole–trimethoprim are both associated with FDE. However, the patient had completed her course of sulfamethoxazole–trimethoprim 2 months prior to this admission. In addition, the FDE was appreciated after the initiation of the phenytoin loading dose and improved after the discontinuation of phenytoin. Thus, we believe these agents are unlikely causes of the FDE.

This case report is unique in that the patient was previously receiving phenytoin for 7 years and although she was subtherapeutic on presentation, there were detectable levels of phenytoin. Appropriate loading and maintenance doses were administered on presentation to attain therapeutic levels.^1,11^ However, the loading dose administration may have been higher than necessary, since the patient was not actively seizing.^11^ Although an appropriate therapeutic concentration was attained, the phenytoin concentrations were not at steady state. The serum concentration that was obtained indicates an appropriate loading dose, but approximately 50 to 75 hours is required to achieve steady state with phenytoin to determine appropriateness of the maintenance dose.^1^

## Conclusions

This case report adds to the current literature of FDE and phenytoin in a patient experiencing an adverse event after long-term treatment with phenytoin. However, this is the first report of a possible association of the development of FDE and the administration rate of phenytoin. If intravenous phenytoin is used, clinicians should follow the current boxed warning that limits the administration of phenytoin to 50 mg/min due to the possibility of adverse reactions and consider FDE in patients regardless of their past exposure to phenytoin.
